# Augmented proximity: Integration of physical and virtual proximity to enhance network connectivity

**DOI:** 10.1371/journal.pone.0260349

**Published:** 2021-11-22

**Authors:** Mi Chang, Ji-Hyun Lee

**Affiliations:** Graduate School of Culture Technology, KAIST, Daejeon, The Republic of Korea; University of Pisa, ITALY

## Abstract

With the continuing increase in online communications, virtual proximity as well as physical proximity has become a common way to connect individuals. Virtual proximity refers to the psychological closeness felt by people based on their participation in a virtual space. Hence, augmenting physical proximity with virtual types is believed to enhance connectivity within social networks, and applications that consider both types have the capability to provide new forms of interaction. However, despite the importance of their coexistence, these proximity concepts have been studied separately or are being integrated using diverse terminologies that often lead to misunderstandings. Furthermore, although some applications reflect the two proximity types (e.g., location-based services), there is no metric of comparison. This paper proposes the concept of augmented proximity (AP), which combines physical and virtual forms into a network analogous to one of mixed reality (MR). The concept provides a clear distinction between physical and virtual proximity using a single quantitative value. Using this concept, a formal taxonomy is established to compare and evaluate AP-based networks. The taxonomy consists of three dimensions that can be analyzed using graph theory, including the extent of connectivity degree, diffusion effect, and extent of perceived closeness. Furthermore, using the services underlying AP-based network, the proposed taxonomy can be applied to evaluate the suitability of the services as an indicator for comparison. The results show that one of the two services has a higher taxonomy-based value, and a reasonable basis for selecting one based on proximity is established. This study suggests that AP will play an important role as a quantitative indicator in developing and comparing applications that consider proximity in both virtual and physical modes.

## Introduction

Social interactions among humans can be viewed in terms of networks. With the continued increase in online communications worldwide, both physical and virtual modes of interacting are being used [1–3]. Research on the coexistence of the two modes has been conducted, resulting in a mixed reality (MR) construct from the perspective of visual displays [4]. MR includes the merger of real and virtual worlds to produce new engagements and visualizations wherein real and virtual objects coexist. It is accompanied by an explanation of the virtuality continuum, which refers to the continuous expanse between the two axes. The space between the two extremes, where reality and virtuality are mixed, is composed of augmented reality and augmented virtuality. This study proposed a formal taxonomy for classifying real and virtual worlds according to three dimensions that included the extent of world knowledge, reproduction fidelity, and the extent of the presence metaphor. The taxonomy offers clear guidelines for distinguishing the various types of MR according to how they are displayed. The MR concept can be summarized using the following three framework components:

- MR definition
- MR virtuality continuum
- MR display taxonomy

The same pattern can be applied to the overall network in which we interact in both physical and virtual modes simultaneously [5]. Hence, no matter how virtually connected people and actors are, their proximities can vary significantly. Therefore, even if the concept of MR is analogous to the network, it must clearly reflect the nature of proximity.

Proximity, defined by the Cambridge Dictionary, is “the state of being near in space or time” [6]. It is a topic that has been studied in fields of human cognition, culture, and emotion. Various types of proximity have been applied to networks. However, with the advent of digital technology, studies have focused primarily on physical and virtual proximity [7, 8].

To investigate the physical proximity of human social groups, Hall introduced proxemics, defined as the identification and analysis of differences in emotional responses to physical distance according to culture [9]. In this context, proximity refers to the physical distance between interacting individuals as influenced by their relationships. Distances and contacts among actors depend on the levels of emotional intimacy between them [10]. Additionally, physical proximity has been analyzed with regard to complex networks by dividing human interactions into short- and long-range interactions, finding that short-range networks can be characterized by densely connected neighborhoods bridged by weak ties [11]. Another study established a new mechanism of segregation to resolve and analyze spatial inequalities and define patterns of spatial proximity based on the connectivity between locations and spatial barriers [12]. Various user applications based on physical proximity have been developed and analyzed [13, 14].

Unlike the conventional notion of proximity, virtual proximity is a modern concept that has arisen with the development of digital technology. An extreme example of virtual proximity is portrayed in the movie *Her* (2013), in which the main character feels love for an artificially intelligent operating system. This fictional example depicts how human emotional proximity can exist in a virtual space [15]. Hence, virtual proximity can be seen to reflect the level of emotional closeness developed through the use of information and communications technology [16]. The emphasis is on the psychological relationship, not the technology. If the local surroundings cannot provide a specific resource (e.g., person or knowledge), virtual proximity can provide opportunities to make use of resources even though they may be physically located far away [17–19]. This paradoxical phenomenon of closeness has been explored with relation to physically distant friends, resulting in a model of perceived proximity. The model shows how communication and social identification processes alongside individual and socio-organizational factors affect feelings of proximity [20]. Virtual proximity facilitates innovation by offering communication and closeness in the virtual space, which play significant roles in higher organizational development [7]. At global-scale companies, virtual proximity is an important method of emphasizing cultural integration for successful work efficiency [21]. Research in the travel field has also highlighted the importance of co-presence given by virtual proximity [22]. For example, a platform called ‘Airbnb’ connects hosts and guests for a short period for renting a house. This platform is generating an economic effect of about a billion dollars for a year in Korea. This result is because it brought local tourism and revitalization of the local economy to places with poor physical proximity [23]. The provision of the platform creates new proximity by overcoming the limitations of physical proximity and increasing virtual proximity. In the field of business investment, virtual proximity offers increased investment connectivity by addressing the inequality of information [24]. Eventually, when virtual proximity is added to physical proximity, connectivity is strengthened within the network. It also helps overcome physical limitations by providing an additional opportunity to the physical environment through an actual application that considers the two proximities.

However, although the coexistence of physical and virtual proximities is important, several limitations have been found in the literature. First, physical and virtual proximity studies were conducted separately. Conceptual definitions and corresponding research that combine the two while acknowledging their relationship are lacking. Notably, mixing concepts and terms without a singular conceptual framework can cause misunderstandings. Second, although there are various ways to express physical proximity in numerical terms, it is not easy to quantify and express virtual proximity, because it primarily reflects psychological concepts. Third, a network based on 2-dimensional proximity already exists (e.g., location-based services and regional community platforms). However, no cases have provided an indicator that provides comparison opportunities of similar services and platforms while identifying physical and virtual proximity.

To overcome these limitations, we propose augmented proximity (AP), which combines the physical and virtual constructs along a single continuum. With AP, we also offer a taxonomy to compare various AP-based networks. Ultimately, the key points can be summarized in three ways similar to MR:

- AP definition
- AP proximity continuum
- AP-based network taxonomy

**Fig 1:**
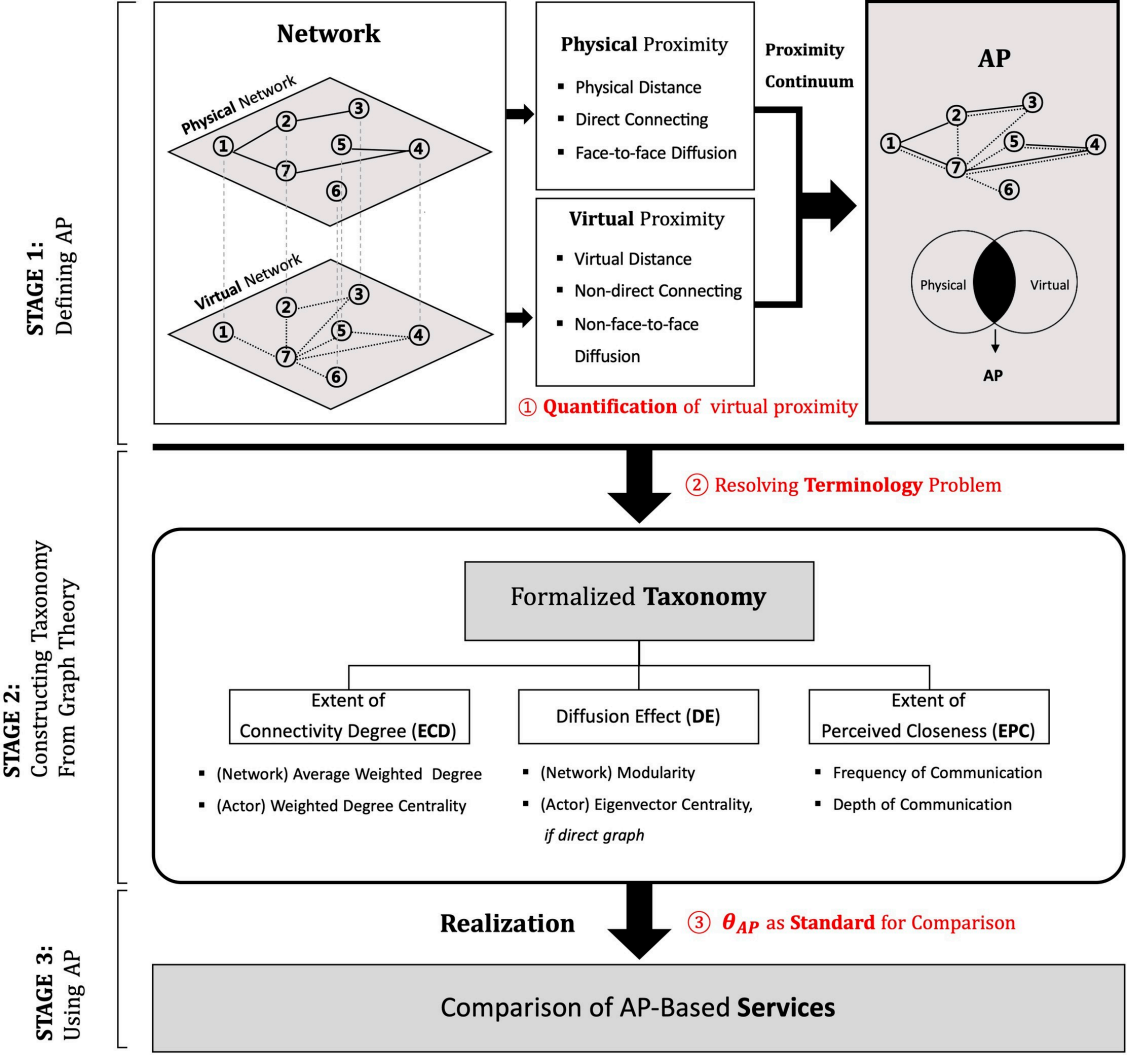
Three-stage process for defining augmented proximity (AP).

This research comprises three stages, as shown in Fig 1. The KAIST Institutional Review Board approved all processes related to this research, and the work was performed in accordance with approved bioethical standards (IRB-21-124). During stage 1, physical and virtual proximity emerge from the physical and virtual network divisions. Each comprises three intersecting factors. Therefore, the two networks can be described by connection weight. During stage 2, a formal taxonomy consisting of three dimensions is provided to allow valid network comparisons and classifications. Each dimension is taken from existing graph theory as a method of network quantification. However, the dimensions are not simply borrowed; instead, their meanings are differentiated according to the two layers mentioned for stage 1, owing to the connections that reflect the AP. During stage 3, we compare the network characteristics with two services underlying the AP-based network, we calculate *θ*_*AP*_ based on the taxonomy, and we quantify the comparison results.

## AP definitions

### Defining the factors

**Fig 2:**
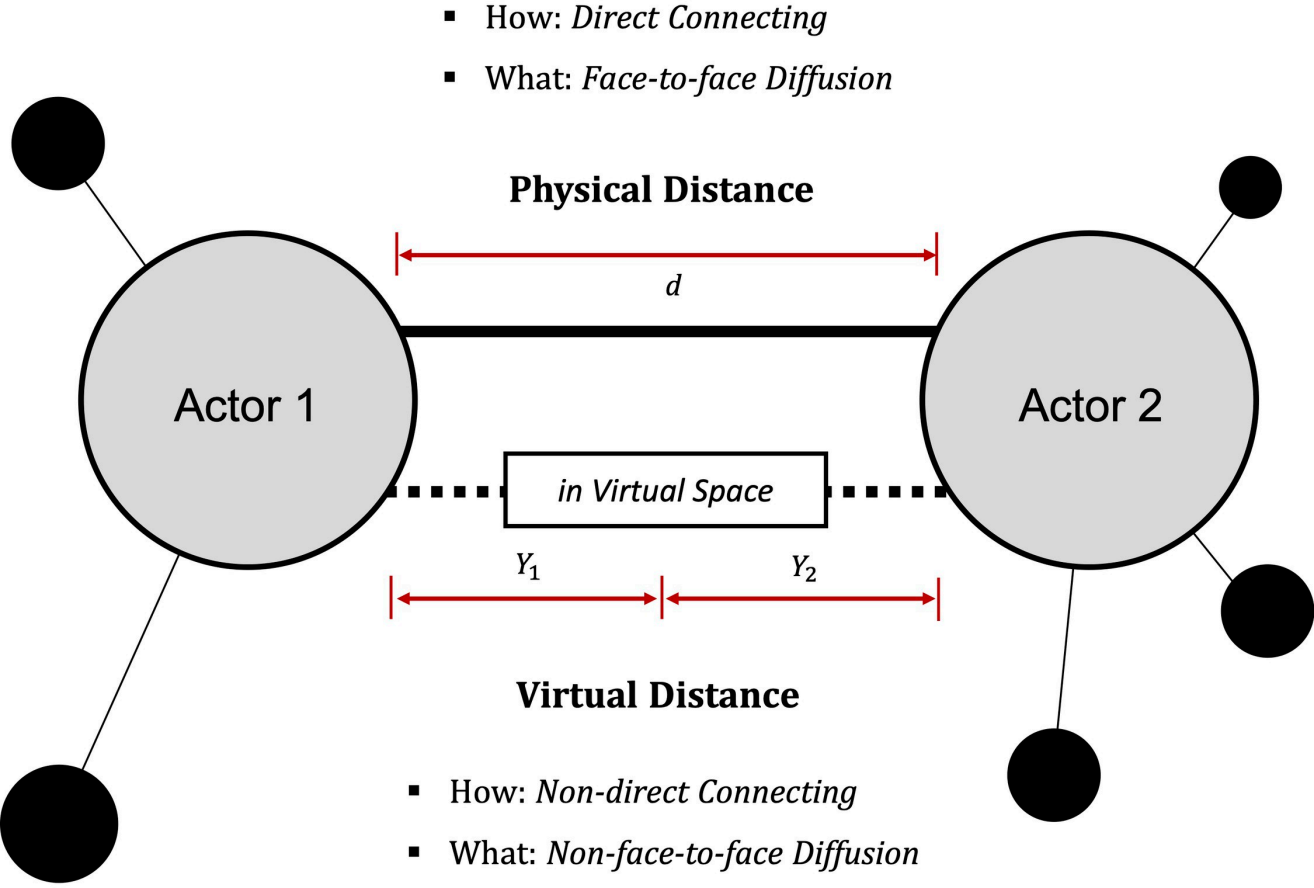
AP factors for distinguishing the physical from the virtual: Physical vs. virtual distance; direct vs. non-direct connecting; and face-to-face vs. non-face-to-face diffusion.

Prior to proposing a new definition, the distinction between the concept of reality and virtuality needs to be clearly presented in terms of MR. From a network perspective, a distinction does exist, but it is used in a variety of ways that differ according to perspective. We distinguish the two types according to three factors shown in Fig 2.

The first factor depends on physical and virtual distances. The following operational definitions are thus adopted:

- Physical distance is the great-circle distance between two points or actors on a sphere.
- Virtual distance is the psychological distance between two actors according to their degree of commitment to the organization, such as one that provides a commercial service, an educational platform, or an information-sharing community in a virtual space.

Physical distance is the geographical distance between two visible and objective points that can be measured quantitatively and accurately [25].

With Eq (1), *d* is the physical distance between actors 1 and 2, where *r* is the radius of the Earth, *ϕ*_1_ and *ϕ*_2_ are the latitudes of the two actors, and *λ*_1_ and *λ*_2_ are the actors’ longitudes.

$$d=2r\sin^{-1}\left(\sqrt{{\text{sin}}^{2}\left(\frac{\phi_{2}-\phi_{1}}{2}\right)}+\cos\left(\phi_{1}\right)\cos\left(\phi_{2}\right){\text{sin}}^{2}\left(\frac{\lambda_{2}-\lambda_{1}}{2}\right)\right).$$ (1)

On the other hand, virtual distance refers to the psychological distance based on the virtual space. The psychological distance refers to the affective, continuance, and normative commitment each actor has toward a specific organization in the virtual space [26]. The organizations may refer to information-sharing communities, educational platforms, or commercial services and can be extended to fit any context. Thus, we specifically describe the three components of commitment, which are important variables in defining virtual distance. First, the *affective* component refers to an actor’s emotional attachment to, identification with, and involvement in the organization. Second, the *continuance* component refers to commitment based on the costs that an actor associates with leaving the organization. Finally, the *normative* component refers to an actor’s feelings of obligation to remain with the organization. These three components become the three variables, *y*_1_, *y*_2_, and y_3_, which define the virtual distance, as in Eq (2), and constitute *Y* by adding *Y*_1_ of actor 1 and *Y*_2_ of actor 2:

$$Y=f\left(y_{1},\;y_{2},\;y_{3}\right)=Y_{1}+Y_{2}.$$ (2)

The second factor depends upon whether there is a connection through the virtual space. In the physical space, direct connections are possible and do not require a virtual space. By carefully distinguishing between direct and non-direct connections and using the appropriate terms, we can reflect differences that occur according to a given method by which a feeling of closeness is achieved.

The final factor is determined between face-to-face and non-face-to-face diffusion. Diffusion within a network has different characteristics, depending on the two conditions. The disease propagation model is an example of face-to-face diffusion [11, 27], whereas information diffusion [28] or marketing motivations are non-face-to-face [29].

By considering these factors, physical and virtual proximity can be clearly differentiated. Physical proximity is expressed as Eq (3) by applying *d* to Eq (1) and the maximum possible physical distance, *d*_*max*_. Normalization is measured from 0 to 100, and as the distance between actors increases, *X* gradually approaches zero. This is taken as the weight of the physical connection between actors, providing a way to quantify physical proximity:

$$X=100\left(-\frac{d}{d_{max}}+1\right).$$ (3)

Virtual proximity is expressed as Eq (4) via linear regression based on three variables of the commitment degree of each actor toward the organization in the virtual world. The characteristics of the proximity in the virtual space are determined according to the coefficients of each variable. Additionally, as with *X*, normalization is performed from 0 to 100, and *Y* becomes the weight of the virtual connection between actors:

$$Y_{1}=\beta_{1}y_{1}+\beta_{2}y_{2}+\beta_{3}y_{3}+\alpha_{1}.$$ (4)

Based on these two types of proximity definitions, we coordinate the AP. Furthermore, we can define the AP via a vector norm that expresses the distance and direction from the origin to a specific point,

$$\dot{Z}=\left(X,Y\right),$$ (5)

$$\|Z\|=\sqrt{X^{2}+Y^{2}}.$$ (6)

Therefore, ‖*Z*‖ is the weight of the connection between actors that represents the AP in the network, and if *X* or *Y* is zero, it is defined as the minimum value of one. Using this value, we can obtain an AP-based network and analyze it with existing graph theory. *Augmented* reflects the proximity of network having a similar context to augmented reality in MR. Combining physical and virtual proximity is not simple. As the proximity as an AP is integrated, the connectivity of the entire network is strengthened, and by providing an AP-based application, it overcomes physical limitations and leads to improvements to the physical value of the region.

### Proximity continuum

MR is described by the virtuality continuum, which ranges between the completely virtual, some virtuality, mostly real, and completely real. Similar patterns and characteristics are seen in networks of physical and virtual worlds. As virtual networks increase, proximity within physical and virtual networks also exists as a continuum. One study investigated whether physical distance affected communications or interactions in a virtual gaming space. Interestingly, homophily and physical proximity was found to influence players’ behavior in the virtual space [30]. As such, physical and virtual proximities affect each other and are inseparable. Further, they are quantified and placed on the continuum in numerical representation. Two proximities must be considered together to understand proximity correctly.

**Fig 3:**
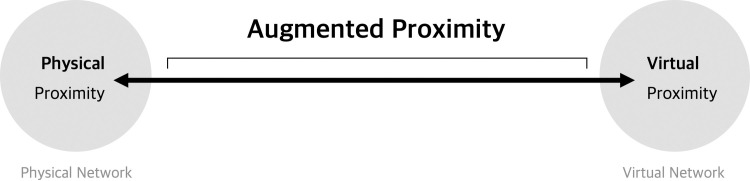
Simplified representation of the proximity continuum, showing AP.

Additionally, physical proximity was found to be an objective indicator, and virtual proximity can be observed via cognitive and subjective indicators derived from human psychology and emotions. The physical and virtual proximities were displayed as extremes from each network. Then, the two proximities combined into one network coexisted on a straight line, as shown in Fig 3, giving us the AP, which is positioned on a straight line according to the degree of physical and virtual proximity. This is the key characteristic of the proximity continuum.

With MR, displays are classified into six classes, from “monitor-based” to “completely graphic” displays along the virtuality continuum. There have yet been no studies in which networks have been classified using the AP. Therefore, we analyzed networks that are commonly classified based on anthropology and personal community [31, 32]. From these, we found five types of networks: family, educational, professional, interest-based, and informational. First, family networks are ones in which there is high physical proximity. Next, educational networks consist of schools or academic organizations that focus on education; as they are formed around the physical spaces of a school. Hence, their characteristics are closer to the physical end of the continuum. Professional networks can be divided into commercial networks (e.g., spaces where consumers and producers meet for commercial purposes) and business networks (e.g., spaces comprising workers that cooperate in various fields centering on work). Interest-based networks are built upon people having the same interests and are closer to the virtual end of the continuum. Finally, informational networks consist of people who share content to obtain answers to various problems in everyday life. Although physical connections sometimes occur, they are highly virtual proximal. The proximity continuum is notably covered by these five network types for which AP already exists.

## AP taxonomy

All network types are based on AP and have distinct features by which physical and virtual proximities intersect. However, although the network types seem to be reasonably and characteristically well-described, the distinctions quickly become blurred when actual networks are applied. This is why MR proposed a formal taxonomy. Although MR display can be divided into six classes by common function, it is quickly blurred when considering concepts such as real, virtual, direct-view, egocentric, and other types. Thus, the intent is to present a taxonomy of principal aspects of MR displays that capture these practical issues.

**Fig 4:**
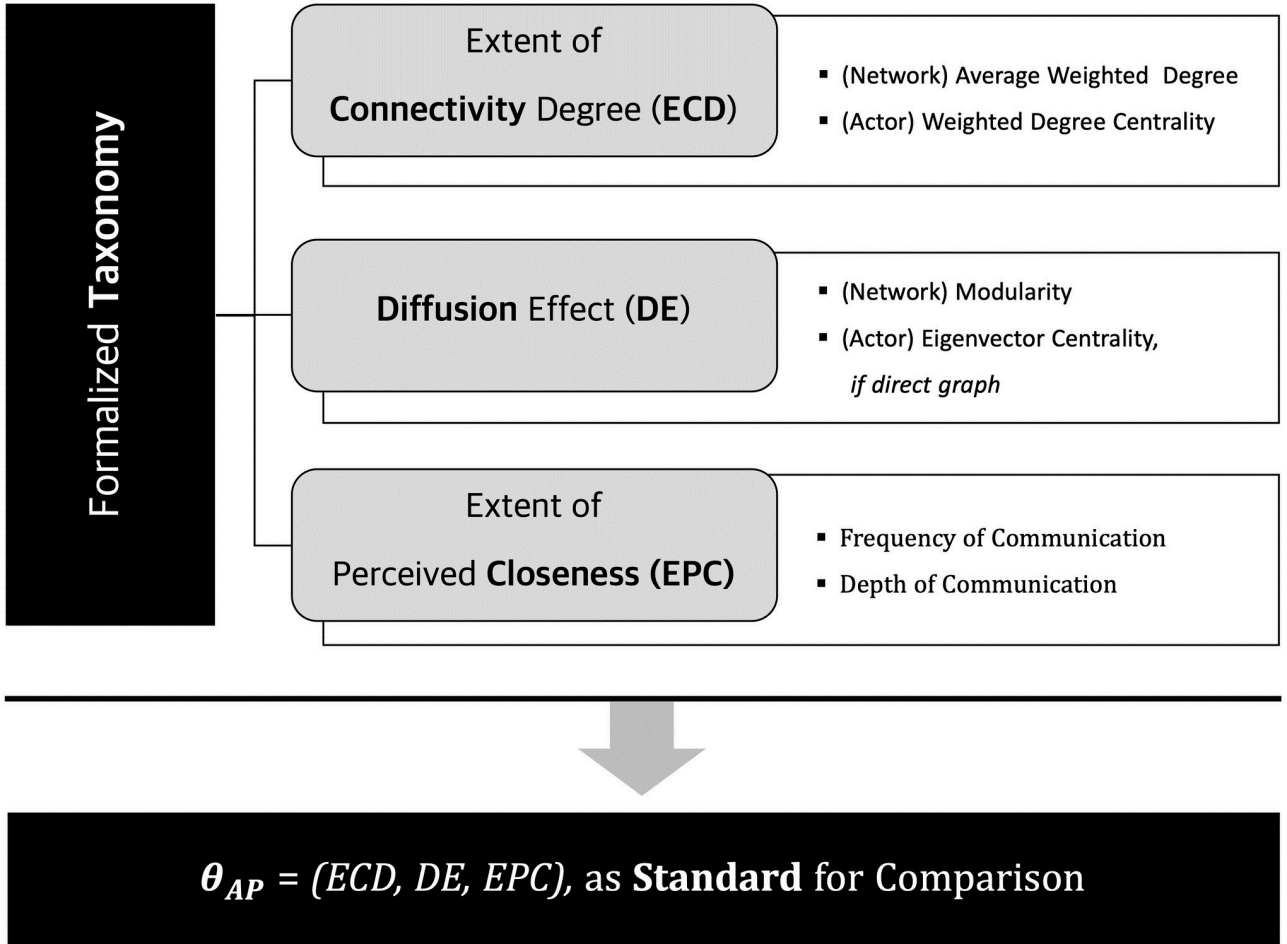
Formalized taxonomy comprising three dimensions: Extent of connectivity degree; diffusion effect; and extent of perceived closeness, and *θ*_*AP*_ as standard for comparison.

Similar to the taxonomy proposed in MR, a taxonomy from a network perspective can be proposed to classify an AP-based network. The purpose of taxonomy is to classify networks according to their contexts and provide criteria for comparing relevant applications. As shown in Fig 4, the formalized taxonomy consists of three dimensions: ECD, DE, and EPC. Because these three dimensions are collectively expressed as *θ*_*AP*_, it can provide an important comparison for applications underlying AP-based networks. Next, we explain each of these dimensions in detail.

### ECD

The importance of the ECD relates to the connectivity within networks. To measure this quantitatively, graph theory is applied. Although it is difficult, because an AP is fused across two layers (i.e., physical and virtual), it can be visualized as a multi-layer structure. Thus, connectivity can be quantified using an average weighted degree and a weighted degree centrality for the actor. Here, average weighted degree is the average of edge weights, and weighted degree centrality reflects the centrality of each node (or actor) by the sum of their edge weights. Although degree centrality does not reflect differences of influence between actors, it is sufficient for expressing connectivity within a single network [33, 34]. Therefore, through the average weighted degree, ECD can be analyzed from the perspective of an entire network, as shown in Eq (7). Furthermore, the actor having the highest centrality can be analyzed through weighted degree centrality. This actor acts as a hub within the network and is classified as the most influential node based on connectivity:

$$ECD=\frac{\sum weights\;of\;edges}{Number\;of\;nodes}.$$ (7)

### DE

The second dimension, DE, is related to the third distinguishing factor between physical and virtual. As a multilayer network is formed, actors interact with resources as needed. Eventually, they are connected via diffusion, which can differ considerably according to the nature of the network and the characteristics of the transmitted elements [35].

Diffusion is closely related to modularity and eigenvector centrality of a network. Modularity is quantitative and is defined as [36] follows:

$$Q=\frac{1}{2m}\underset{i,j}{\sum }\left[A_{ij}-\frac{k_{i}k_{j}}{2m}\right]\delta \left(c_{i},c_{j}\right),$$ (8)

where *A*_*ij*_ represents the weight of the edge between *i* and *j*, *k*_*i*_ = Σ_*j*_*A*_*ij*_ is the sum of the weights of the edges attached to vertex *i*, *c*_*i*_ is the community to which vertex *i* is assigned, the *δ* function *δ(u*, *v)* is one if *u = v* and zero otherwise, and $m=\frac{1}{2}\underset{ij}{\sum }A_{ij}$.

This is an important metric for analyzing how and with what density links within the community connect. The effect of diffusion is deeply influenced by the composition and characteristics of the community. Moreover, the role and influence of the hub within the community causes a spread to occur at a rapid pace, owing to the network’s modularity [37]. Therefore, *Q*, which represents the degree of modularity, becomes an index representing diffusion from the perspective of the entire network:

DE=Q. (9)

Although a modularized network may allow spread to occur readily within the community, it does not easily occur between communities. In particular, entities that diffuse through physical contact, such as a virus, are sometimes transmitted between communities by *familiar strangers* outside the community [11]. Therefore, it is necessary to analyze for actors that cause this diffusion. When using a directed graph, eigenvector centrality values can be used to quantitatively analyze the diffusion effect of modularity. Eigenvector centrality is a measure of the influence of actors in the network; connections to actors that score high on this metric contribute more to spreading within a network than do low-scoring actors [38].

### EPC

The third dimension of the taxonomy is EPC, which is the degree to which actors perceive each other within a network. To include this factor, we must recognize that virtual proximity can be considered alongside physical proximity. Even if both physical and virtual proximity exists in the same network, their values among actors can differ, and this factor can be used to classify AP more cognitively. Thus, perceived proximity characteristics of communication [20], which increases with the frequency and depth of communication, influence the perception of proximity through three mechanisms: increasing cognitive salience, reducing uncertainty, and envisioning another’s context. These mechanisms describe how an actor identifies others, which includes adding them to one’s own social category through a common ground.

EPC is divided into frequency of communication and depth of communication. A high frequency of communication increases cognitive importance even over physical distance. Cognitive importance refers to how easily and how often an issue or another person comes to mind [39]. With frequent communication, even someone who is physically far away can be at the top of a person’s mind, thus appearing psychologically closer. A study investigating a social network based on the exchange of Christmas cards found that the frequency of communication was deeply related to emotional closeness [40]. Thus, the higher the level of emotional closeness between individuals, the shorter the time since their last contact. Thus, high emotional closeness increases the frequency of communication, and this holds for AP-based networks.

Depth of communication, on the other hand, is closely related to the five levels of communication [41]. The first level is *ritual*, which is a simple form of interaction that allows two people to recognize each other as human beings that exchange short greetings and similar courtesies. It serves as the foundation for deepening interpersonal relationships. The second level is *extended ritual*. For example, a person may have a brief conversation with the professional cleaner seen daily, but topics of conversation can change. This is a safe level of communication and helps develop trust. The third level is *content*, which is a professional interaction that does not include discussion about emotions. The fourth level is *feelings about content*, which are exchanged when there is sufficient safety and trust. The last level is *feelings about each other*, which are expressed as one of the most direct forms of interpersonal feedback. By referring to these five levels, depths of connection within an AP-based network can be described.

As shown in Eq (10), *a* is the frequency of communication, and *b* is the depth, expressed as *(a*, *b)*. The norm of the two is calculated as the EPC:

$$EPC=\sqrt{a^{2}+b^{2}}.$$ (10)

**Fig 5:**
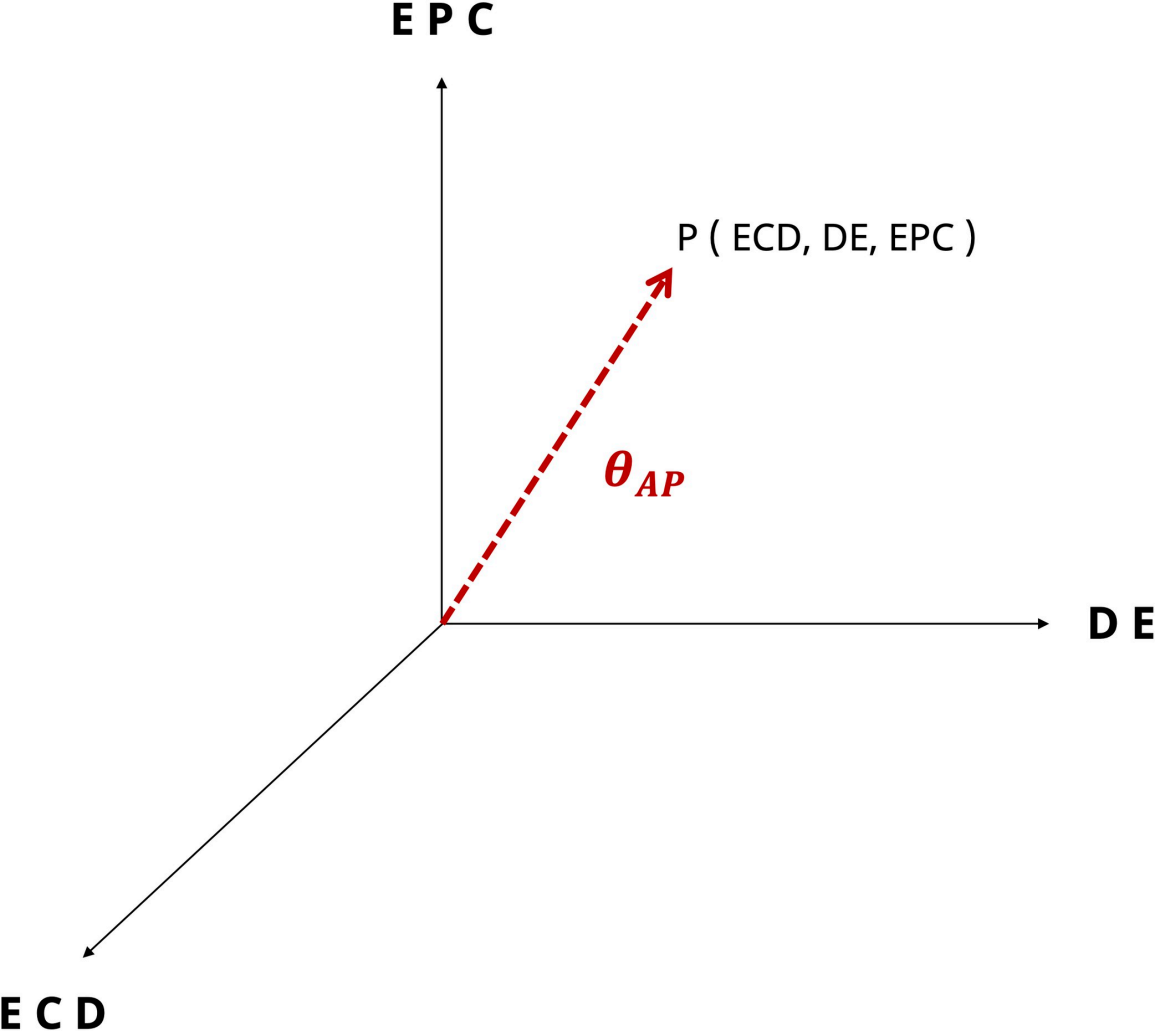
Description of *θ*_*AP*_ represented as a single value through a three-dimensional taxonomy.

*ECD*, *DE*, and *EPC* are equally normalized from zero to one and are then calculated as a norm, shown in Eq (11) and Fig 5, and expressed as one indicator: *θ*_*AP*_.


$$\theta_{AP}=\sqrt{{\left(ECD\right)}^{2}+{\left(DE\right)}^{2}+{\left(EPC\right)}^{2}}.$$ (11)

Since the maximum value of each dimension is 1, the maximum value is √3 (≈1.732). Here, *θ*_*AP*_ quantifies the AP-related network characteristics consisting of three axes from 0 to 1.732, making it possible to compare the networks.

### Using AP

AP-based networks can be extended to location-based services and information-sharing communities. To clarify, we compare and analyze two services underlying AP-based networks to provide examples. Thus, we use Danggeun Market and Slack networks, which have the highest number of downloads in South Korea among mobile applications having AP characteristics. Danggeun Market allows users to directly trade used goods in the Dong area of Korea. Otherwise, they may not trade. Slack, on the other hand, allows students to share work statuses and hold meetings by forming a research community. Because Slack communities are usually organized around projects, members of the same school comprise a majority, but researchers from other affiliations can also participate. Although these two services are AP-based networks, they have different characteristics, which can show distinct differences in proximity and provide clear explanations. The Danggeun Market is called service 1 and Slack is called service 2.

The survey was conducted from a total of 14 participants who used both services. The questionnaire retrieved residential addresses and user perceptions of the two services (S1 Appendix). Regarding user perceptions, the survey was conducted on a 7-point scale through questions about commitments proposed by *Allen* [26] to apply Eq (2) previously described.

The physical distance, *d*, was calculated using the latitude and longitude values of each actor’s residence. Service 1 occurred only within a small area (i.e., Dong) as verified by location authentication. To calculate the physical proximity, *d*_*max*_ was set to 20 km. In service 2, because all users in Korea were connected through the scholarly project, the longest possible distance in Korea was 500 km, which was set as *d*_*max*_. Therefore, physical proximity between actors was calculated using Eq (3).

To calculate the virtual distance, *Y*, based on the survey, the coefficients were obtained as part of three components, *affective*, *continuance*, and *normative*. Through Eq (4) described previously, the survey results of each component were coordinated and linear regression was performed. The coefficients were calculated using *scikit-learn*, a library provided by *Python*. As a result of linear regression, Eq (12) was used for service 1, and Eq (13) was used for service 2.

$$Y_{service1}=0.25y_{1}+0.26y_{2}+0.16y_{3}+1.17,$$ (12)

$$Y_{service2}=0.34y_{1}+0.26y_{2}+0.30y_{3}+0.40,$$ (13)

It was therefore possible to measure the virtual distance of each actor for services 1 and 2. Additionally, the virtual distances of the two actors were summed to calculate the virtual proximity. This value is defined as virtual proximity, *Y*, through a normalization process from 0 to 100.

**Fig 6:**
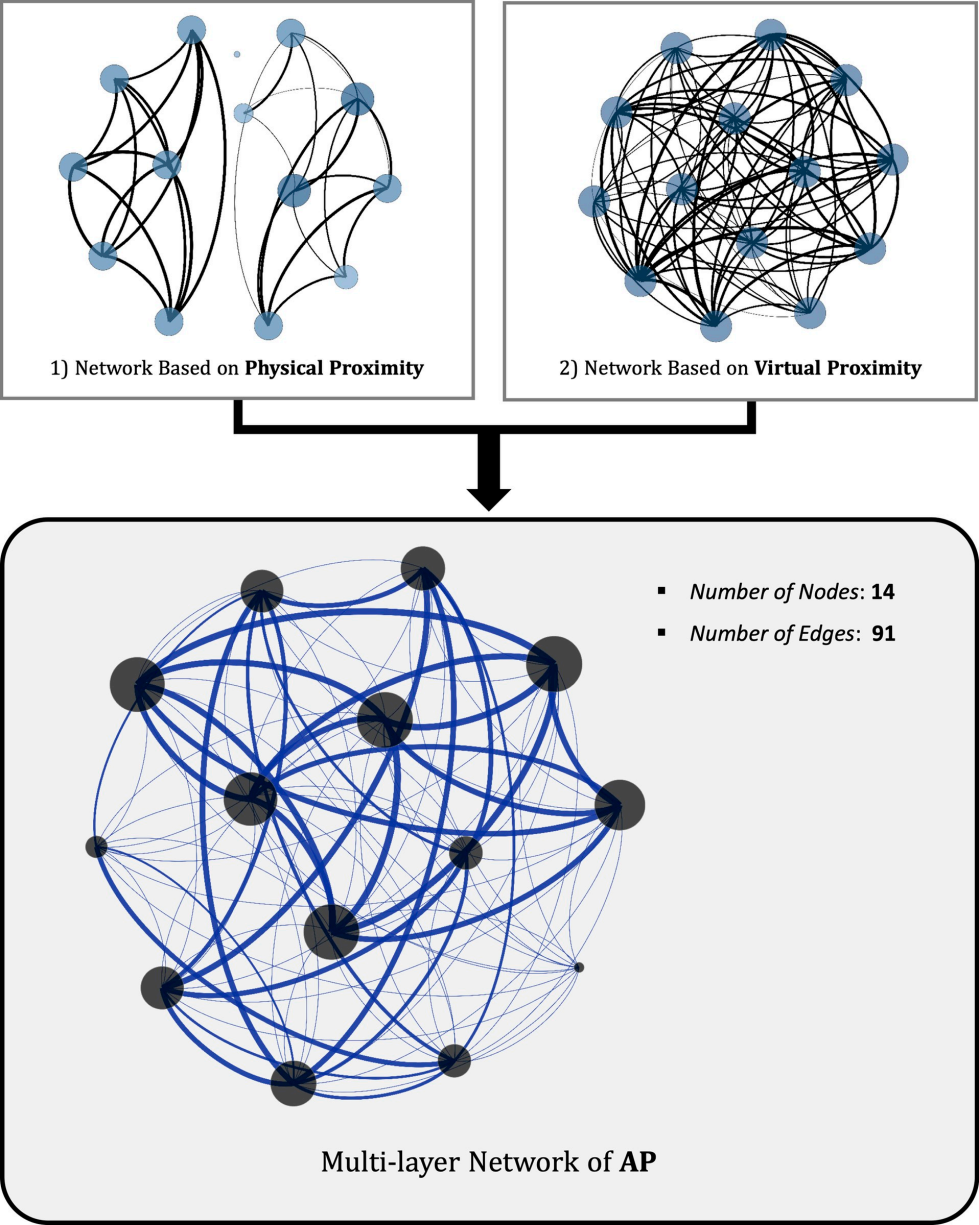
Visual representation of augmented-proximity (AP)-based multi-layer network through convergence of physical- and virtual-proximity networks based on service 1.

The calculated physical and virtual proximities became the weights of the edges in each network. To analyze the AP-based network, two types of the proximity were calculated as the norm, and the AP was obtained. The AP was defined as the weight of the edge in the AP-based network. Fig 6 shows the network visualization of Service 1. The two networks at the top were applied for physical and virtual proximity, and the bottom was an AP-based network in which two layers were combined.

First, based on physical proximity, the network was divided into several components, because the *d*_*max*_ was set as low as 20 km; thus, there were cases in which there was no connection between actors. Then, there were cases in which actors were outliers. Second, in a virtual proximity-based network, because a survey was conducted with people who used service 1, it had one component. However, edges have different weights depending on the degree of virtual proximity. Finally, based on the AP-based network, it was identified as one component, and it can be seen that the weight of the edge was clearly different depending on the AP degree.

**Fig 7:**
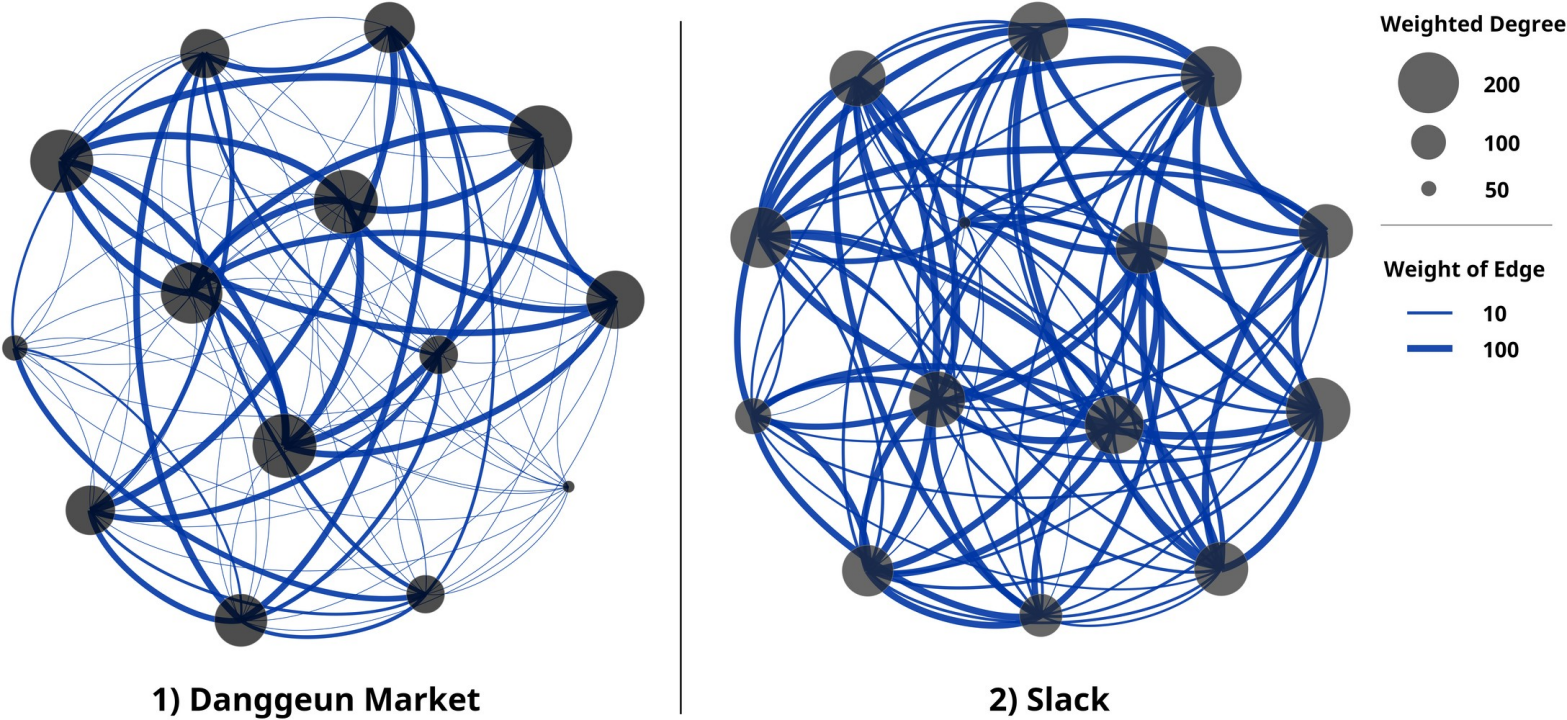
Augmented-proximity (AP)-based network visualization that reflects weight according to AP characteristics based on two services.

Based on proximity-related characteristics, services 1 and 2 can be visualized as AP-based networks, respectively, as shown in Fig 7. Using the three dimensions of taxonomy and *θ*_*AP*_ as shown in Table 1, it is possible to compare and analyze the two services.

**Table 1:** Measured values of three dimensions of taxonomy and *θ*_*AP*_ in two services with augmented-proximity (AP) characteristics.

	Measured value	Service 1	Service 2
		(Danggeun Market)	(Slack)
**Dimension 1 of taxonomy**	Average weighted degree	355.478	1,171.49
	ECD	0.267	0.781
**Dimension 2 of taxonomy**	Modularity	0.470	0.018
	DE	0.470	0.018
**Dimension 3 of taxonomy**	$\sqrt{a^{2}+b^{2}}$	1.567	3.490
	EPC	0.142	0.622
***θ***_***AP***_		0.559	0.998

With ECD, the first dimension, service 2, which has a significantly higher *d*_*max*_ has a higher value than service 1. In the second dimension, the value of service 1 was 0.470, which is higher than the 0.018 of service 2. This is a numerical value affected by the physical proximity. For service 1, when *d*_*max*_ exceeded 20 km, the weight of the edge decreased, and the reduced weight had a great influence on community formation. In the third dimension, service 2, which reflected frequent communications between actors and relatively deep conversations, was calculated as the higher value. Reflecting these characteristics, *θ*_*AP*_ was calculated, which was an important indicator used to evaluate services. The *θ*_*AP*_ of service 1 was 0.559, and the *θ*_*AP*_ of service 2 was 0.998. Service 2 has the *θ*_*AP*_ of about 1.78 times compared to service 1. As mentioned previously, this value is an indicator that can select the dominant network in AP formation by expressing the taxonomy as a single value. This numerical result shows that service 2 has dominant network characteristics in AP formation compared to service 1. We compared services with different characteristics to show clear differences, but there are times when it is necessary to select one service from among the services of the same domain based on proximity. For example, we have a restaurant that needs publicity. For promotion, we need to choose a platform that is easy to reach our customers, and it is difficult to intuitively know which platform is the most appropriate. In this case, *θ*_*AP*_ allows us to select a platform that is advantageous for AP formation, which has the potential to reach customers more easily than other platforms.

In the end, when comparing services through the calculated *θ*_*AP*_, it helps to select an advantageous service in terms of AP by considering the physical and the virtual network at the same time. Furthermore, time and economic waste can be reduced by selecting a service that can more easily reach users through *θ*_*AP*_.

## Conclusion

The coexistence of virtual and physical proximity is important to social experiences because humanity is on the verge of ubiquitous near-real-time communications with anyone at any time. Merging the physicality and virtuality of such experiences is essential to bringing the society closer together. However, the two types of proximity have been studied separately or have been combined through terms from different perspectives that can be misleading. Furthermore, no metric exists to compare applications based on two proximity-based networks. To overcome these limitations, we introduced the term “augmented proximity” or “AP,” which combines physical and virtual proximity onto a continuum within a single network. A quantification method was proposed by analyzing factors that define physical and virtual proximity in a network. AP applies the weights of edges between actors in the network. A formal taxonomy was then proposed to classify various applications within the AP-based network. ECD, DE, EPC definitions were provided, expressed as the 3-dimensional term, *θ*_*AP*_, which enables clear comparisons among networks.

Three contributions are presented in this article. First, the proposed AP construct reflects the proximity of a network comprising both physical and virtual worlds. It solves terminology issues plaguing extant relationship models that describe physical and virtual proximities. Furthermore, the AP-based network was analyzed from the network perspective using existing graph theory. Second, the psychological factors related to virtual proximity were quantified based on the actors’ commitment to the organization in the virtual world. The quantified virtual proximity was integrated into physical proximity, making it possible to quantify as an AP. This AP value gave a new meaning to the edge weight in the multi-layer network. Third, we proposed a taxonomy-based indicator that allows applications having similar characteristics to be compared according to their purpose. Using this numerical value, rational choices can be made considering the proximity of actors within the physical and virtual worlds.

This study is currently limited to theoretical constructs. To overcome this limitation, our theoretical construction will be extended to secondary research, which will be applied to real-world and computationally optimized. We plan to express a specific region of South Korea as an AP-based network. Regions are increasingly connected in virtual as well as physical ways with technological advances [42]. Using these characteristics, we will study solutions to increase the number of visitors and revitalize the local economy by analyzing traffic data and social media data in a specific area with an AP-based network.

## Supporting information

S1 Appendix User survey to compare two services based on AP. (PDF)

S1 File Table of edge weight for the AP-based network. (XLSX)
