# Supplementary material for: Augmented proximity: Integration of physical and virtual proximity to enhance network connectivity
Source: PLoS One. 2021 Nov 22;16(11):e0260349. doi: 10.1371/journal.pone.0260349 (PMC8608320; doi:10.1371/journal.pone.0260349)
Supplement: S1 Appendix — (PDF) [file pone.0260349.s001.pdf]

S1 Appendix. User Survey to Compare Two Services based on AP

1. Purpose

- This survey was conducted to compare and analyze two services underlying AP-based networks

2. Process

- This survey took on average about 20 minutes. Participants had experience using the service, and it was conducted for 14 participants. Due to COVID-19, all processes were conducted online through the delivery of a survey link.
- The reward for participation was 10,000 won (about 9 dollars).

3. Questions

- The survey questions were modified according to the characteristics of the service while maintaining the structure proposed by *Allen* [26].

<u>1. Demographic data collection</u>

1) Please select your gender (male / female)

2) Please select your age group (20s / 30s)

3) Please select your occupation (student / housewife / office worker / other)

4) Please enter your current residence address up to 'Dong'

<u>2. Data collection related to the service characteristics</u>

2-1. Whether to use the service

1) Have you ever used the Danggeun Market service?

: (Yes / No)

2) Have you ever used the Slack service?

: (Yes / No)

2-2. Commitments

- Repeat the following questions about 1) Danggeun Market and 2) Slack service.

1) **Affective** Commitment Scale Items

(a) I would be very happy to spend my life with this service

①	②	③	④	⑤	⑥	⑦

Not very ... *Very much*

(b) I enjoy discussing events taking place within this service with people

①	②	③	④	⑤	⑥	⑦

Not very ... *Very much*

(c) I really feel as if this service's problem are my own

①	②	③	④	⑤	⑥	⑦
Not very						*Very much*

(d) I think that I could easily become as attached to another service as I am to this one (R)

①	②	③	④	⑤	⑥	⑦
Not very						*Very much*

(e) This service has a great deal of personal meaning for me

①	②	③	④	⑤	⑥	⑦
Not very						*Very much*

2) **Continuance** Commitment Scale Items

(a) It would be very hard for me to leave this service right now, even if I wanted to

①	②	③	④	⑤	⑥	⑦
Not very						*Very much*

(b) Too much in my life would be disrupted if I decided I wanted to leave this service now

①	②	③	④	⑤	⑥	⑦
Not very						*Very much*

(c) Right now, staying with this service is a matter of necessity as much as desire

①	②	③	④	⑤	⑥	⑦
Not very						*Very much*

(d) I feel that I have too few options to consider leaving this service

①	②	③	④	⑤	⑥	⑦
Not very						*Very much*

(e) One of the few serious consequences of leaving this service would be the scarcity of available alternatives

①	②	③	④	⑤	⑥	⑦
Not very						*Very much*

3) **Normative** Commitment Scale Items

(a) I think services come and go too often these days.

①	②	③	④	⑤	⑥	⑦
Not very						*Very much*

(b) One of the major reasons I continue to use for this service is that I believe that loyalty is important and therefore feel a sense of moral obligation to remain

①	②	③	④	⑤	⑥	⑦
Not very						*Very much*

(c) If there is a chance to move to a better service, I would not feel it was right to leave this service

①	②	③	④	⑤	⑥	⑦
Not very						*Very much*

(d) I was taught to believe in the value of remaining loyal to one service

①	②	③	④	⑤	⑥	⑦
Not very						*Very much*

(e) Things were better in the days when people stayed with one domain and one service.

①	②	③	④	⑤	⑥	⑦

Not very *Very much*

2-3. Virtual Proximity

(a) How close do you feel emotionally while using this service?

①	②	③	④	⑤	⑥	⑦

Not very close *very close*
